# Inaugural Editorial, January 2023

**DOI:** 10.1093/molbev/msac265

**Published:** 2023-01-05

**Authors:** Brandon S Gaut, Claudia A M Russo

**Affiliations:** UC Irvine, Department of Ecology and Evolutionary Biology, 321 Steinhaus Hall, Irvine, CA; Departamento de Genetica, Universidade Federal do Rio de Janeiro, Rio de Janeiro, Brazil

This year marks the 40th year of publication of *Molecular Biology and Evolution* (MBE). Over the past four decades, the importance of molecular evolution as a research field has grown substantially, encouraged by advances in sequencing technology, new kinds of -omics data, enhanced computational power, and commensurate advances in theories and protocols. The discipline of molecular evolution remains central for understanding the history of life, the processes that shape the diversity of life, and the functional significance of evolutionary change. Our field also has more practical relevance than ever before, because it provides foundational insights into critical societal challenges like the history and spread of pandemics, the evolutionary causes and consequences of human disease, and the biological ramifications of climate change from a molecular viewpoint. Through the decades of change, MBE has kept pace with the remarkable advancements in our field by offering a venue to publish high-quality work that explores evolutionary hypotheses.

As the field has expanded, so has our community. MBE relies on the dedicated service of more than 60 Editorial Board members who volunteer their time and expertise. But community involvement does not stop there: over the past 2 years, the journal utilized the expertise of over 6,000 reviewers to improve the manuscripts of nearly 5,000 authors. Publishing in MBE benefits this extensive community directly, because MBE is an open access, not-for-profit journal. Although MBE relies on author publication charges to fund its editorial and publishing operations, all remaining funds are returned to the Society (SMBE). These funds underwrite SMBE activities, including annual conferences, numerous satellite meetings, and individual fellowships and awards.

As the newly appointed co Editors-in-Chief of MBE, we are honored and privileged to serve this vibrant research community. Our foremost goal is to continue to provide a venue for the publication of high-quality science. During our term, we also plan to prioritize initiatives that will broadly serve and strengthen our community, by lowering barriers to publication of quality science and by promoting cooperation and inclusivity among evolutionary biologists.

Good science should be published irrespective of the authors” ability to pay. We ask authors who do not have funds for article processing charges to submit a waiver request. All waiver decisions for MBE and our sister journal, *Genome Biology and Evolution* (GBE), are now handled by an SMBE committee that is independent of the editorial boards. We intend to strengthen further our relationship with GBE by streamlining the process of transferring manuscripts between journals. In the future, we hope to share additional initiatives with GBE and with other not-for-profit, society-owned journals.

Among our first actions, we have made the MBE submission process more flexible and straightforward, and we have also initiated a pilot project to consider revisions of previous submissions to other journals with their accompanying reviews. The motivation for considering revised manuscripts from outside journals alongside prior reviews is to lessen the reviewing burden on the scientific community, while ensuring that MBE publishes only high-quality science. We have been exploring additional improvements to MBE, including expanding our social media presence and strengthening our publishing pipelines for Reviews and Protocols, because we believe these have the potential to especially benefit our community. We also hope to find ways to reward reviewers for their services and continue to build an editorial board that reflects the growing diversity of our community as a whole.

We cannot close without extending a special thank you to Sudhir Kumar, who has been the Editor-in-Chief for the last decade. Sudhir has served the SMBE community with unusual distinction, and his considerable efforts have contributed to MBE's current strong reputation in our field. During his tenure, the journal increased both the number of submissions and the Impact Factor, which remained strong for 2021 at 8.8. In addition to overseeing the journal, Sudhir has served as SMBE president and organized two SMBE annual meetings. The entire SMBE community owes him our heartfelt gratitude.

Publishing in MBE contributes to its distinguished legacy and benefits *our* scientific community. As members of that community, we are always happy to hear your ideas and to know how we can better serve your needs. Please also join us to celebrate MBE's 40th year this July in Ferrara, Italy for the first in-person SMBE meeting since 2019!

